# Simple Organics and Biomonomers Identified in HCN Polymers: An Overview

**DOI:** 10.3390/life3030421

**Published:** 2013-07-29

**Authors:** Marta Ruiz-Bermejo, María-Paz Zorzano, Susana Osuna-Esteban

**Affiliations:** 1 Departamento de Evolución Molecular, Centro de Astrobiología (CSIC-INTA), Ctra, Torrejón-Ajalvir, km 4, 28850-Torrejón de Ardoz, Madrid, Spain; E-Mail: osunaes@cab.inta-csic.es; 2 Departamento Instrumentación Avanzada, Centro de Astrobiología (CSIC-INTA), Ctra, Torrejón-Ajalvir, km 4, 28850-Torrejón de Ardoz, Madrid, Spain; E-Mail: zorzanomm@cab.inta-csic.es

**Keywords:** HCN polymers, prebiotic synthesis, nucleobases, amino acids, carboxylic acids, chromatographic techniques

## Abstract

Hydrogen cyanide (HCN) is a ubiquitous molecule in the Universe. It is a compound that is easily produced in significant yields in prebiotic simulation experiments using a reducing atmosphere. HCN can spontaneously polymerise under a wide set of experimental conditions. It has even been proposed that HCN polymers could be present in objects such as asteroids, moons, planets and, in particular, comets. Moreover, it has been suggested that these polymers could play an important role in the origin of life. In this review, the simple organics and biomonomers that have been detected in HCN polymers, the analytical techniques and procedures that have been used to detect and characterise these molecules and an exhaustive classification of the experimental/environmental conditions that favour the formation of HCN polymers are summarised. Nucleobases, amino acids, carboxylic acids, cofactor derivatives and other compounds have been identified in HCN polymers. The great molecular diversity found in HCN polymers encourages their placement at the central core of a plausible protobiological system.

## 1. Introduction

Different strategies for the prebiotic synthesis of biomonomers have been designed assuming that the reactive precursors [*i.e.*, hydrogen cyanide (HCN), formaldehyde (CH_2_O), formamide (HCONH_2_), ammonium cyanide (NH_4_CN), cyanoacetylene and others] were available in adequate concentrations on the primitive Earth or elsewhere [1]. The prebiotic transformation most likely occurred in water (oceans, lagoons or lakes) at moderate pH values or in solid-state conditions caused by the slow evaporation of water.

HCN (also known as prussic acid) is considered to be one of the most important and versatile building blocks for the construction of biomolecules. HCN was prepared for the first time by C. W. Scheele (1742–1786). He heated blood with KOH and charcoal and obtained what he called “Blutlage”, which he distilled with sulphuric acid [2]. The oligomerisation of HCN was first observed by J. L. Proust in 1806 [3]. In 1874, R. Wipperman published the results of his studies on the conversion of aqueous HCN into its trimer (aminomalonic acid dinitrile), which subsequently hydrolysed and decarboxylated to produce glycine, which he separated by crystallisation and identified by elemental analysis [4]. Only one year later, E. Pflüger published one of the earliest chemical speculations concerning the origin of “living proteins” from cyano compounds: “cyanogen and its compounds had plenty of time and opportunity to follow their great tendency to transformation and polymerisation and, by the addition of oxygen and later water and salts, to change to a labile protein, which constitutes living matter” [5]. Almost one century later, J. Oró claimed the first prebiotic synthesis of adenine from refluxed solutions of concentrated ammonium cyanide [6]. Since then, HCN polymerisation has generally been considered the preferential prebiotic route for the synthesis of purines and pyrimidine derivatives. Thus, it has been suggested that HCN polymers may be important substances in the first stages of the chemical evolution of life.

From an astrobiological point of view, HCN is a very interesting reactant. HCN molecules and CN radicals are ubiquitous in the Universe. This molecule is observed in planetary and interstellar locations and is easily produced in plausible prebiotic environments. HCN has been detected in remote galaxies [7], in interstellar clouds [8,9], notably in star-forming regions [10,11,12,13,14], in reflection nebulae [15,16,17,18], in planetary nebulae [19], in interplanetary dust [20,21], in circumstellar envelopes and discs [22,23,24,25,26,27], in comets [28,29,30,31], in meteorites [32], in the atmospheres of the outer planets and their moons [33,34,35,36,37] and, in a terrestrial context, in volcanic gases and hydrothermal vents [38,39]. HCN is the major product when appropriate gas mixtures are subjected to intense physical energy, such as from electric discharges, UV radiation or shock waves [40,41,42,43,44].

HCN can spontaneously polymerise in the presence of bases, such as ammonia and free radicals from ionising radiation, and the spontaneous polymerisation occurs over a wide range of temperatures and pressures in both polar (water) and non-polar (hydrocarbon) solvents and surfaces. Recently, a synthesis has also described the formation of an HCN polymer from formamide [45]. The HCN polymers, also known as HCN oligomers, azulmic acid or azulmin, are heterogeneous solids ranging in colour from yellow or orange to brown or black, depending on the degrees of polymerisation and cross-linking. The structures of HCN polymers have not been fully characterised and remain controversial due to their complex and heterogeneous nature. Several models try to explain the complex structure of HCN polymers [46,47,48,49,50,51,52]. However, the structure, nature and behaviour of HCN polymers are intriguing issues, and although some aspects have been resolved, many questions remain unanswered. Active research into the nature and properties of the HCN polymers is still in progress [53,54,55,56,57].

A number of studies were carried out over the last fifty years to define the molecules present in the HCN polymers, and excellent reviews have been published addressing this topic [58,59,60,61]. Nevertheless, comparing the products obtained under different reaction conditions or different analytical procedures is difficult. In this review, an updated and comprehensive catalogue of the simple organics and biomonomers found in the HCN polymers is shown. The experimental/environmental conditions for the polymerisation of HCN are described for each compound as well as the analytical techniques and procedures that should be used to detect and characterise them. Additionally, some mechanistic aspects are described, particularly for the production of nucleobases.

## 2. Amino Acids

In 1953, S. L. Miller demonstrated with his famous and successful experiment that a prebiotic formation of amino acids was possible [62]. After this pioneering work, J. Oró investigated the possible role of the aqueous polymerisation of HCN in the amino acids production [63]. Glycine, alanine and aspartic acid were identified by paper chromatography after heating a concentrated ammoniacal solution of HCN at 70 °C for 25 days. On the basis of this experiment, many other conditions have been used to investigate the HCN polymerisation processes, which can be grouped into four rough categories: (i) heating experiments; (ii) room temperature experiments; (iii) radiation experiments; (iv) freezing experiments. In the heating experiments, ammoniacal solutions of HCN (2.2–0.1 M) are heated at 100–38 °C for 1–30 days. In the room temperature experiments, similar solutions of HCN are used (1–0.1 M), but the reaction times are increased from 1 month to 18 months. It is well known that only concentrated solutions of HCN (>0.01 M)) can polymerise and produce nucleic-acid bases and amino acids, whereas in dilute solutions, hydrolysis becomes dominant [64]. Taking into account the production rates of HCN in the primitive atmosphere and the experimental hydrolysis rates, the steady state concentration of HCN in the primitive ocean could be in the range of 4 × 10^–6^−2 × 10^–8^ M at a pH value of 8 between 0 °C and 25 °C [65]. Miyakawa *et al*. [66] estimated this concentration to be approximately 2 × 10^–6^ M at pH = 8 and 0 °C. Therefore, if HCN polymerisation was actually important for the production of the first and essential biomolecules, there must have been routes by which diluted HCN solutions were efficiently concentrated. Because HCN is more volatile than water, it cannot be concentrated by evaporation if the pH is lower than the pK_a_ of HCN (9.2 at 25 °C). Therefore, an alternative and plausible mechanism is eutectic freezing. Thus, using freezing conditions, Miller and co-workers achieved the synthesis of glycine, alanine and aspartic acid from a frozen ammonium cyanide solution (HCN 0.1 M plus NH_3_ 0.1 M, pH 9.2) that had been held at from −20 to −78 °C for periods of 2 months to 25 years [67]. The eutectic phase of an aqueous HCN solution, which occurs at −23.4 °C, contains 74.5% HCN (25 M) [64]. In an early terrestrial context, because eutectic formation requires complete freezing, it is possible to propose the shallowest pools or areas under the most severe glacier conditions as more favourable sites for HCN oligomerisation. Additionally, it is possible to suggest other planetary objects in the Solar Systems that have these conditions, such as the icy moons of Jupiter [67]. The characteristics of eutectic systems merit further investigation in the contexts of chemical evolution and planetary exploration, in particular, in the context of the next mission to Europa, Ganymede and Callisto (JUICE mission, 2020).

Moreover, ionising radiation can also catalyse the polymerisation of HCN. Among the available sources of energy that may have enabled chemical evolution in the early Earth were UV radiation and radioactivity [68]. Abelson showed the production of glycine, alanine, aspartic acid and serine using diluted solutions of HCN at pH 8–9 and UV radiation [69]. Draganic and co-workers have studied the amino acids formed by the acid hydrolysis of oligomers from ^60^Co-radiolysis of HCN [70,71].

**Figure 1:**
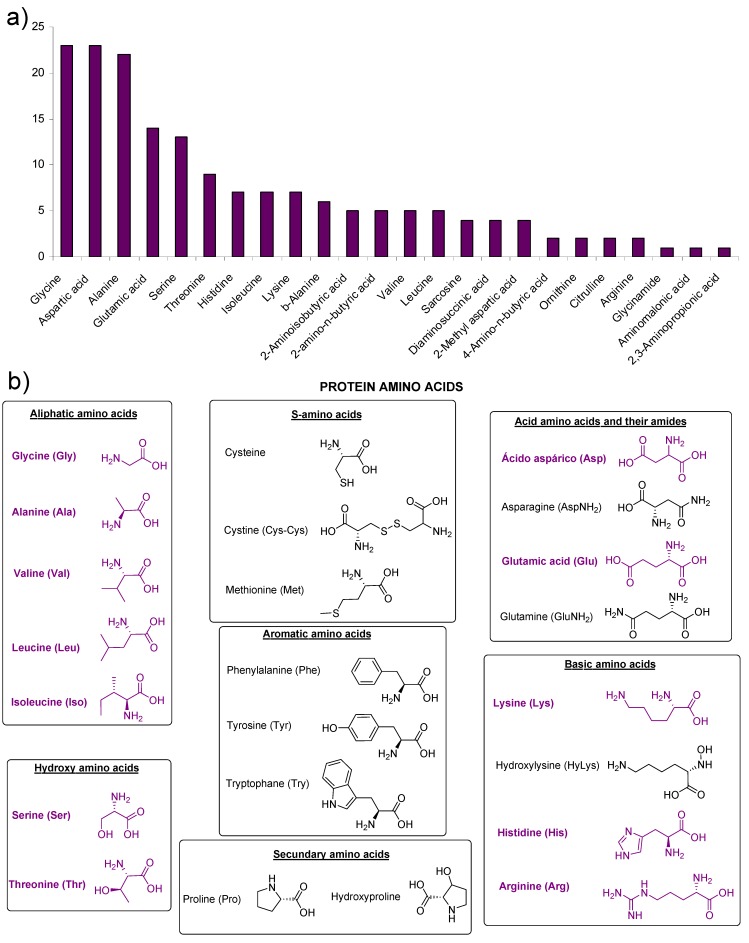
(**a**) Amino acids detected in Hydrogen Cyanide (HCN) polymers; (**b**) The protein amino acids found in HCN polymers are shown in colour.

Table 1 describes the conditions of synthesis and hydrolysis for HCN polymers and the analytical methods for the identification of the amino acids. One can see that most of the experiments were performed in aqueous solution, and only a few experiments were carried out under anhydrous conditions. Using the four groups of conditions described, twenty four amino acids were identified (see Figure 1a). In most cases, acid hydrolysis of the HCN polymers was necessary before their detection and identification. The HCN polymers yield twelve of the twenty protein amino acids (Figure 1b). It is interesting to note that glycine and aspartic acid are the only amino acids that can be identified under all conditions assayed (twenty three sets of experimental conditions). Moreover, glycine is the amino acid that is always obtained with the greatest yield. Labadie *et al*. estimated a glycine yield of approximately 0.11 mol/L [72]. In contrast, glycinamide, aminomalonic acid and 2,3-aminopropionic acid were detected under unique reaction conditions. Sarcosine and 2-methyl aspartic acid have only been detected under γ-radiation conditions and 4-amino-n-butyric acid and ornithine under hot conditions at 90 °C. Diaminosuccinic acid was only found in room temperature experiments by Ferris and co-worker (Figure 1a).

Taking into consideration that most of the experiments shown in Table 1 were carried out between 1961 and 1984 and great developments in chromatography and analytical techniques have taken place since that time, further identification of the organic compounds in HCN polymers may be necessary. The identification of amino acids seems to depend on the experimental conditions of the polymerisation, the sample preparation and the analytical tools used. The discovery of alkaline lakes or soda lakes with pH values between 9 and 12 presents a new possibility for the concentration of cyanide in tidal beaches by evaporation and encourages a revision of the experiments at moderate temperatures and relatively high concentration of cyanide. The mechanism for the formation of amino acids from HCN polymerisation has not been described, but it has been suggested that the trimer (aminomalononitrile, AMN) or tetramer (diaminomaleonitrile, DAMN) of HCN might be involved in the synthesis. Studies of the formation of amino acids from AMN and DAMN were conducted by Matthews and co-worker [73,74] as well as Ferris and co-worker [64]. 

**Table 1:** Amino acids identified in Hydrogen Cyanide (HCN) polymers. The reaction conditions are described together with the material analysed: soluble fraction (solution or soluble oligomers), insoluble fraction (brown or dark precipitates) or raw reaction. c (M) = initial molar concentration of the reactant in aqueous solution; HCN_(L)_ = HCN in liquid phase; HCN_(G)_ = HCN in gas phase; d = days; m = moths; y = years;Non hydrolysis = no additional hydrolysis (acid, basic or neutral) was made over the final product analysed; Acid = HCl 6N/100–110 °C/16–24 h; Basic = NaOH 0.1 N/100 °C/6 h; Neutral = NaOH pH 8–8.5/110 °C/6–24 h; GC-MS = Gas Chromatography-Mass Spectrometry; AAA = Automatic amino acid analyzer; PC = paper chromatography; HPLC = High performance liquid chromatography.

Compound	Starting material, c (M)	T (°C)/t/Catalyst	Final product analysed	Hydrolysis	Method of identification	Reference
Glycine	HCN, 0.2	100/1 d/-	Raw mixture	Acid	GC-MS	[75]
	NH_4_CN, 1	90/4 h/-	Brown precipitate	Acid	AAA	[76]
	HCN, 1	90/4 h/NH_4_OH (pH 9.58)	Black solid	Acid	2D-PC	[72]
	HCN, 1.5	90/18 h/NH_3_	Soluble oligomers	Acid	2D-PC, AAA	[77]
	HCN, 1.5	70/5 d/NH_4_OH	Soluble oligomers	Non hydrolysis	PC	[78]
	HCN, 2.2	70/25 d/NH_4_OH	Soluble oligomers	Non hydrolysis	PC	[63]
	NaCN, 1	38/3–30 d/NH_4_Cl	Black solid	Acid	GC-MS	[46]
	HCN_(L)_	r.t./4 w/anhydrous NH_3_	Dark solid residue	Acid	AAA	[51]
	NaCN, 1	r.t./3 m/pH 9.2 (HCl)	Soluble oligomers	Acid, basic and neutral	GC-MS	[79]
	HCN, 0.1	r.t./1–6 m/NH_4_OH (pH 9.2)	Soluble oligomers	Acid	AAA, GC-MS	[80,81]
	HCN, 0.1	r.t./4–12 m/NH_4_OH (pH 9.2)	Soluble oligomers	Acid and neutral	GC-MS	[82]
	HCN, 0.1	r.t./18 m/NH_4_OH (pH 9.2)	Soluble oligomers	Acid, Basic and neutral	GC-MS	[79]
	HCN, 0.002–0.1	r.t./UV radiation (Hg lamp)/pH (8–9)	Soluble oligomers	Acid	AAA	[69]
	HCN_(G)_	r.t./UV radiation (Hg lamp)	Solid	Acid	AAA	[83]
	HCN, 0.004–0.1	r.t./γ-radiation (^60^Co source)	Raw mixture	Acid	PC, AAA, GC-MS	[84]
	HCN, 0.1	r.t./γ-radiation (^60^Co source)/pH 6	Raw mixture	Acid	AAA, GC-MS	[70]
	HCN, 0.1	r.t./γ-radiation (^60^Co source)/NH_3_/pH 9	Solution	Acid	AAA, GC-MS	[70]
	NaCN, 0.1	r.t./γ-radiation (^60^Co source)/pH 11.3	Solution	Acid	AAA, GC-MS	[70]
	HCN, 0.1	r.t.−40 °C/γ-radiation (^60^Co source)/NH_3_/pH 9	Solution	Acid	AAA, GC-MS	[71]
	HCN, 1.5	Refrigerator/4 d/NH_3_	Black Solid	Acid	AAA	[51]
	HCN, 0.1	−20/2 m/NH_3_/pH 9.2	Solution	Acid	HPLC	[67]
	HCN, 0.1	−20/25 y/NH_3_/pH 9.2	Solution	Acid	HPLC	[67]
	HCN, 0.1	−78/25 y/NH_3_/pH 9.2	Solution	Acid	HPLC	[67]
Glycinamide	HCN, 1.5	70/5 d/NH_4_OH	Soluble oligomers	Non hydrolysis	PC	[78]
Aminomalonic acid	NaCN, 1	38/3–30 d/NH_4_Cl	Black solid	Acid	GC-MS	[46]
Alanine	HCN, 1.5	100/1 d/-	Raw mixture	Acid	GC-MS	[75]
	NH4CN, 1	90/4 h/-	Brown precipitate	Acid	AAA	[76]
	HCN, 1	90/4 h/NH_4_OH (pH 9.58)	Black solid	Acid	2D-PC	[72]
	HCN, 1.5	90/18 h/NH_3_	Soluble oligomers	Acid	2D-PC, AAA	[77]
	HCN, 1.5	70/5 d/NH_4_OH	Soluble oligomers	Non hydrolysis-	PC	[78]
	HCN, 2.2	70/25 d/NH_4_OH	Soluble oligomers	Non hydrolysis	PC	[63]
	HCN_(L)_	r.t./4 w/anhydrous NH_3_	Dark solid residue	Acid	AAA	[51]
	NaCN, 1	r.t./3 m/pH 9.2 (HCl)	Soluble oligomers	Acid, basic and neutral	GC-MS	[79]
	HCN, 0.1	r.t./1–6 m/NH_4_OH (pH 9.2)	Soluble oligomers	Acid	AAA, GC-MS	[80,81]
	HCN, 0.1	r.t./4–12 m/NH_4_OH (pH 9.2)	Soluble oligomers	Acid and neutral	GC-MS	[82]
	HCN, 0.1	r.t./18 m/NH_4_OH (pH 9.2)	Soluble oligomers	Acid, Basic and neutral	GC-MS	[79]
	HCN, 0.002–0.1	r.t./UV radiation (Hg lamp)/pH (8–9)	Solution	Acid	AAA	[69]
	HCN_(G)_	r.t./UV radiation (Hg lamp)	Solid	Acid	AAA	[83]
	HCN, 0.004–0.1	r.t./γ-radiation (^60^Co source)	Raw mixture	Acid	PC, AAA, GC-MS	[84]
	HCN, 0.1	r.t./γ-radiation (^60^Co source)/pH 6	Raw mixture	Acid	AAA, GC-MS	[70]
	HCN, 0.1	r.t./γ-radiation (^60^Co source)/NH_3_/pH 9	Solution	Acid	AAA, GC-MS	[70]
	NaCN, 0.1	r.t./γ-radiation (^60^Co source)/pH 11.3	Solution	Acid	AAA, GC-MS	[70]
	HCN, 0.1	r.t.−40 °C/γ-radiation (^60^Co source)/NH_3_/pH 9	Solution	Acid	AAA, GC-MS	[71]
	HCN, 1.5	Refrigerator/4 d/NH_3_	Black Solid	Acid	AAA	[51]
	HCN, 0.1	−20/2 m/NH_3_/pH 9.2	Solution	Acid	HPLC	[67]
	HCN, 0.1	−20/25 y/NH_3_/pH 9.2	Solution	Acid	HPLC	[67]
	HCN, 0.1	−78/25 y/NH_3_/pH 9.2	Solution	Acid	HPLC	[67]
β-alanine	HCN, 1.5	90/18 h/NH_3_	Soluble oligomers	Acid	2D-PC, AAA	[77]
	HCN, 0.1	r.t./4–12 m/NH_4_OH (pH 9.2)	Soluble oligomers	Acid and neutral	GC-MS	[82]
	HCN, 0.1	r.t./γ-radiation (^60^Co source)/pH 6	Raw mixture	Acid	AAA, GC-MS	[70]
	HCN, 0.1	r.t./γ-radiation (^60^Co source)/NH_3_/pH 9	Solution	Acid	AAA, GC-MS	[70]
	NaCN, 0.1	r.t./γ-radiation (^60^Co source)/pH 11.3	Solution	Acid	AAA, GC-MS	[70]
	HCN, 0.1	r.t.−40 °C/γ-radiation (^60^Co source)/NH_3_/pH 9	Solution	Acid	AAA, GC-MS	[71]
Sarcosine	HCN, 0.1	r.t./γ-radiation (^60^Co source)/pH 6	Raw mixture	Acid	AAA, GC-MS	[70]
	HCN, 0.1	r.t./γ-radiation (^60^Co source)/NH_3_/pH 9	Solution	Acid	AAA, GC-MS	[70]
	NaCN, 0.1	r.t./γ-radiation (^60^Co source)/pH 11.3	Solution	Acid	AAA, GC-MS	[70]
	HCN, 0.1	r.t.−40 °C/γ-radiation (^60^Co source)/NH_3_/pH 9	Solution	Acid	AAA, GC-MS	[71]
Serine	NH4CN, 1	90/4 h/-	Brown precipitate	Acid	AAA	[76]
	HCN, 1	90/4 h/NH_4_OH (pH 9.58)	Black solid	Acid	2D-PC	[72]
	HCN, 1.5	90/18 h/NH_3_	Soluble oligomers	Acid	2D-PC, AAA	[77]
	HCN_(L)_	r.t./.4 w/anhydrous NH_3_	Dark solid residue	Acid	AAA	[51]
	HCN, 0.1	r.t./1–6 m/NH_4_OH (pH 9.2)	Soluble oligomers	Acid	AAA	[80,81]
	HCN, 0.002–0.1	r.t./UV radiation (Hg lamp)/pH (8–9)	Solution	Acid	AAA	[69]
	HCN_(G)_	r.t./UV radiation (Hg lamp)	Solid	Acid	AAA	[83]
	HCN, 0.004	r.t./γ-radiation (^60^Co source)	Raw mixture	Acid	PC, AAA, GC-MS	[84]
	HCN, 0.1	r.t./γ-radiation (^60^Co source)/pH 6	Raw mixture	Acid	AAA	[70]
	HCN, 0.1	r.t./γ-radiation (^60^Co source)/NH_3_/pH 9	Solution	Acid	AAA	[70]
	NaCN, 0.1	r.t./γ-radiation (^60^Co source)/pH 11.3	Solution	Acid	AAA	[70]
	HCN, 0.1	r.t.−40 °C/γ-radiation (^60^Co source)/NH_3_/pH 9	Solution	Acid	AAA, GC-MS	[71]
	HCN, 1.5	Refrigerator/4 d/NH_3_	Black Solid	Acid	AAA	[51]
2,3-Aminopropioinic acid	HCN, 1.5	90/18 h/NH_3_	Soluble oligomers	Acid	2D-PC, AAA	[77]
Aspartic acid	HCN, 0.2	100/1 d/-	Raw mixture	Acid	GC-MS	[75]
	NH_4_CN, 1	90/4 h/-	Brown precipitate	Acid	AAA	[76]
	HCN, 1	90/4 h/NH_4_OH (pH 9.58)	Black solid	Acid	2D-PC	[72]
	HCN, 1.5	90/18 h/NH_3_	Soluble oligomers	Acid	2D-PC, AAA	[77]
	HCN, 1.5	70/5 d/NH_4_OH	Soluble oligomers	Non hydrolysis	PC	[78]
	HCN, 2.2	70/25 d/NH_4_OH	Soluble oligomers	Non hydrolysis	PC	[63]
	NaCN, 1	38/3–30 d/NH_4_Cl	Black solid	Acid	GC-MS	[46]
	HCN_(L)_	r.t./4 w/anhydrous NH_3_	Dark solid residue	Acid	AAA	[51]
	NaCN, 1	r.t./3 m/pH 9.2 (HCl)	Soluble oligomers	Acid, basic and neutral	GC-MS	[79]
	HCN, 0.1	r.t./1–6 m/NH_4_OH (pH 9.2)	Soluble oligomers	Acid	AAA, GC-MS	[80,81]
	HCN, 0.1	r.t./4–12 m/NH_4_OH (pH 9.2)	Soluble oligomers	Acid and neutral	GC-MS	[82]
	HCN, 0.1	r.t./18 m/NH_4_OH (pH 9.2)	Soluble oligomers	Acid, Basic and neutral	GC-MS	[79]
	HCN, 0.002–0.1	r.t./UV radiation (Hg lamp)/pH (8–9)	Solution	Acid	AAA	[69]
	HCN_(G)_	r.t./UV radiation (Hg lamp)	Solid	Acid	AAA	[83]
	HCN, 0.004–0.1	r.t./γ-radiation (^60^Co source)	Raw mixture	Acid	PC, AAA, GC-MS	[84]
	HCN, 0.1	r.t./γ-radiation (^60^Co source)/pH 6	Raw mixture	Acid	AAA, GC-MS	[70]
	HCN, 0.1	r.t./γ-radiation (^60^Co source)/NH3/pH 9	Solution	Acid	AAA, GC-MS	[70]
	NaCN, 0.1	r.t./γ-radiation (^60^Co source)/pH 11.3	Solution	Acid	AAA, GC-MS	[70]
	HCN, 0.1	r.t.−40 °C/γ-radiation (^60^Co source)/NH_3_/pH 9	Solution	Acid	AAA, GC-MS	[71]
	HCN, 1.5	Refrigerator/4 d/NH_3_	Black Solid	Acid	AAA	[51]
	HCN, 0.1	−20/2 m/NH_3_/pH 9.2	Solution	Acid	HPLC	[67]
	HCN, 0.1	−20/25 y/NH_3_/pH 9.2	Solution	Acid	HPLC	[67]
	HCN, 0.1	−78/25 y/NH_3_/pH 9.2	Solution	Acid	HPLC	[67]
Diaminosuccinic acid	NaCN, 1	r.t./3 m/pH 9.2 (HCl)	Soluble oligomers	Acid, basic and neutral	GC-MS	[79]
	HCN, 0.1	r.t./1–6 m/NH_4_OH (pH 9.2)	Soluble oligomers	Acid	AAA, GC-MS	[80,81]
	HCN, 0.1	r.t./4–12 m/NH_4_OH (pH 9.2)	Soluble oligomers	Acid and neutral	GC-MS	[82]
	HCN, 0.1	r.t./18 m/NH_4_OH (pH 9.2)	Soluble oligomers	Acid, Basic and neutral	GC-MS	[79]
2-aminoisobutyric acid	HCN, 0.2	100/1 d/-	Raw mixture	Acid	GC-MS	[75]
	NaCN, 1	r.t./3 m/pH 9.2 (HCl)	Soluble oligomers	Acid, basic and neutral	GC-MS	[79]
	HCN, 0.1	r.t./1–6 m/NH_4_OH (pH 9.2)	Soluble oligomers	Acid	GC-MS	[80,81]
	HCN, 0.1	r.t./4–12 m/NH_4_OH (pH 9.2)	Soluble oligomers	Acid and neutral	GC-MS	[82]
	HCN, 0.1	r.t./18 m/NH_4_OH (pH 9.2)	Soluble oligomers	Acid, Basic and neutral	GC-MS	[79]
2-amino-n-butyric acid	HCN, 1.5	90/18 h/NH_3_	Soluble oligomers	Acid	2D-PC, AAA	[77]
	HCN, 0.1	r.t./γ-radiation (^60^Co source)/pH 6	Raw mixture	Acid	AAA, GC-MS	[70]
	HCN, 0.1	r.t./γ-radiation (^60^Co source)/NH_3_/pH 9	Solution	Acid	AAA, GC-MS	[70]
	NaCN, 0.1	r.t./γ-radiation (^60^Co source)/pH 11.3	Solution	Acid	AAA, GC-MS	[70]
	HCN, 0.1	r.t.−40 °C/γ-radiation (^60^Co source)/NH_3_/pH 9	Solution	Acid	AAA, GC-MS	[71]
4-amino-n-butyric acid	NH4CN, 1	90/4 h/-	Brown precipitate	Acid	AAA	[76]
	HCN, 1	90/4 h/NH_4_OH (pH 9.58)	Black solid	Acid	2D-PC	[72]
Threonine	NH4CN, 1	90/4 h/-	Brown precipitate	Acid	AAA	[76]
	HCN, 1	90/4 h/NH_4_OH (pH 9.58)	Black solid	Acid	2D-PC	[72]
	HCN, 1.5	90/18 h/NH_3_	Soluble oligomers	Acid	2D-PC, AAA	[77]
	HCN_(L)_	r.t./4 w/anhydrous NH_3_	Dark solid residue	Acid	AAA	[51]
	HCN, 0.1	r.t./1–6 m/NH_4_OH (pH 9.2)	Soluble oligomers	Acid	AAA	[80,81]
	HCN_(G)_	r.t./UV radiation (Hg lamp)	Solid	Acid	AAA	[83]
	HCN, 0.004–0.1	r.t./γ-radiation (^60^Co source)	Raw mixture	Acid	PC, AAA, GC-MS	[84]
	HCN, 0.1	r.t.−40 °C/γ-radiation (^60^Co source)/NH_3_/pH 9	Solution	Acid	AAA, GC-MS	[71]
	HCN, 1.5	Refrigerator/4 d/NH_3_	Black Solid	Acid	AAA	[51]
Glutamic acid	HCN, 0.2	100/1 d/-	Raw mixture	Acid	GC-MS	[75]
	NH4CN, 1	90/4 h/-	Brown precipitate	Acid	AAA	[76]
	HCN, 1.5	90/18 h/NH_3_	Soluble oligomers	Acid	2D-PC, AAA	[77]
	HCN_(L)_	r.t./4 w/anhydrous NH_3_	Dark solid residue	Acid	AAA	[51]
	NaCN, 1	r.t./3 m/pH 9.2 (HCl)	Soluble oligomers	Acid, basic and neutral	GC-MS	[79]
	HCN, 0.1	r.t./1–6 m/NH_4_OH (pH 9.2)	Soluble oligomers	Acid	AAA, GC-MS	[80,81]
	HCN, 0.1	r.t./18 m/NH_4_OH (pH 9.2)	Soluble oligomers	Acid, Basic and neutral	GC-MS	[79]
	HCN_(G)_	r.t./UV radiation (Hg lamp)	Solid	Acid	AAA	[83]
	HCN, 0.004–0.1	r.t./γ-radiation (^60^Co source)	Raw mixture	Acid	PC, AAA, GC-MS	[84]
	HCN, 0.1	r.t./γ-radiation (^60^Co source)/pH 6	Raw mixture	Acid	AAA, GC-MS	[70]
	HCN, 0.1	r.t./γ-radiation (^60^Co source)/NH_3_/pH 9	Solution	Acid	AAA, GC-MS	[70]
	NaCN, 0.1	r.t./γ-radiation (^60^Co source)/pH 11.3	Solution	Acid	AAA, GC-MS	[70]
	HCN, 0.1	r.t.−40 °C/γ-radiation (^60^Co source)/NH_3_/pH 9	Solution	Acid	AAA, GC-MS	[71]
	HCN, 1.5	Refrigerator/4 d/NH_3_	Black Solid	Acid	AAA	[51]
2-methyl aspartic acid	HCN, 0.1	r.t./γ-radiation (^60^Co source)/pH 6	Raw mixture	Acid	AAA, GC-MS	[70]
	HCN, 0.1	r.t./γ-radiation (^60^Co source)/NH_3_/pH 9	Solution	Acid	AAA, GC-MS	[70]
	NaCN, 0.1	r.t./γ-radiation (^60^Co source)/pH 11.3	Solution	Acid	AAA, GC-MS	[70]
	HCN, 0.1	r.t.−40 °C/γ-radiation (^60^Co source)/NH_3_/pH 9	Solution	Acid	AAA, GC-MS	[71]
Ornithine	NH4CN, 1	90/4 h/-	Brown precipitate	Acid	AAA	[76]
	HCN, 1	90/4 h/NH^4^OH (pH 9.58)	Black solid	Acid	2D-PC	[72]
Histidine	NH4CN, 1	90/4 h/-	Brown precipitate	Acid	AAA	[76]
	HCN, 1	90/4 h/NH^4^OH (pH 9.58)	Black solid	Acid	2D-PC	[72]
	HCN_(L)_	r.t./4 w/anhydrous NH_3_	Dark solid residue	Acid	AAA	[51]
	HCN, 0.1	r.t./1–6 m/NH_4_OH (pH 9.2)	Soluble oligomers	Acid	AAA	[80,81]
	HCN_(G)_	r.t./UV radiation (Hg lamp)	Solid	Acid	AAA	[83]
	HCN, 0.1	r.t.−40 °C/γ-radiation (^60^Co source)/NH_3_/pH 9	Solution	Acid	AAA, GC-MS	[71]
	HCN, 1.5	Refrigerator/4 d/NH_3_	Black Solid	Acid	AAA	[51]
Valine	HCN_(L)_	r.t./4 w/anhydrous NH_3_	Dark solid residue	Acid	AAA	[51]
	HCN, 0.1	r.t./1–6 m/NH_4_OH (pH 9.2)	Soluble oligomers	Acid	AAA	[80,81]
	HCN_(G)_	r.t./UV radiation (Hg lamp)	Solid	Acid	AAA	[83]
	HCN, 0.1	r.t./γ-radiation (^60^Co source)	Raw mixture	Acid	PC, AAA, GC-MS	[84]
	HCN, 1.5	Refrigerator/4 d/NH_3_	Black Solid	Acid	AAA	[51]
Isoleucine	HCN, 1.5	90/18 h/NH_3_	Soluble oligomers	Acid	2D-PC, AAA	[77]
	HCN (L)	r.t./4 w/anhydrous NH_3_	Dark solid residue	Acid	AAA	[51]
	NaCN, 1	r.t./3 m/pH 9.2 (HCl)	Soluble oligomers	Acid, basic and neutral	GC-MS	[79]
	HCN, 0.1	r.t./1–6 m/NH_4_OH (pH 9.2)	Soluble oligomers	Acid	AAA, GC-MS	[80,81]
	HCN, 0.1	r.t./18 m/NH_4_OH (pH 9.2)	Soluble oligomers	Acid, Basic and neutral	GC-MS	[79]
	HCN_(G)_	r.t./UV radiation (Hg lamp)	Solid	Acid	AAA	[83]
	HCN, 1.5	Refrigerator/4 d/NH_3_	Black Solid	Acid	AAA	[51]
Leucine	HCN, 1.5	90/18 h/NH_3_	Soluble oligomers	Acid	2D-PC, AAA	[77]
	HCN (L)	r.t./4 w/anhydrous NH_3_	Dark solid residue	Acid	AAA	[51]
	HCN, 0.1	r.t./1–6 m/NH_4_OH (pH 9.2)	Soluble oligomers	Acid	AAA	[80,81]
	HCN_(G)_	r.t./UV radiation (Hg lamp)	Solid	Acid	AAA	[83]
	HCN, 1.5	Refrigerator/4 d/NH_3_	Black Solid	Acid	AAA	[51]
Citrulline	HCN, 0.1	r.t./1–6 m/NH_4_OH (pH 9.2)	Soluble oligomers	Acid	AAA	[80,81]
	HCN, 0.1	r.t.−40 °C/γ-radiation (^60^Co source)/NH_3_/pH 9	Solution	Acid	AAA, GC-MS	[71]
Lysine	NH4CN, 1	90/4 h/-	Brown precipitate	Acid	AAA	[76]
	HCN, 1	90/4 h/NH_4_OH (pH 9.58)	Black solid	Acid	2D-PC	[72]
	HCN_(L)_	r.t./4 w/anhydrous NH_3_	Dark solid residue	Acid	AAA	[51]
	HCN, 0.1	r.t./1–6 m/NH_4_OH (pH 9.2)	Soluble oligomers	Acid	AAA	[80,81]
	HCN_(G)_	r.t./UV radiation (Hg lamp)	Solid	Acid	AAA	[83]
	HCN, 0.1	r.t.−40 °C/γ-radiation (^60^Co source)/NH_3_/pH 9	Solution	Acid	AAA, GC-MS	[71]
	HCN, 1.5	Refrigerator/4 d/NH_3_	Black Solid	Acid	AAA	[51]
Arginine	HCN_(L)_	r.t./4 w/anhydrous NH_3_	Dark solid residue	Acid	AAA	[51]
	HCN, 1.5	Refrigerator/4 d/NH_3_	Black Solid	Acid	AAA	[51]

### Hydantoins

**Figure 2:**
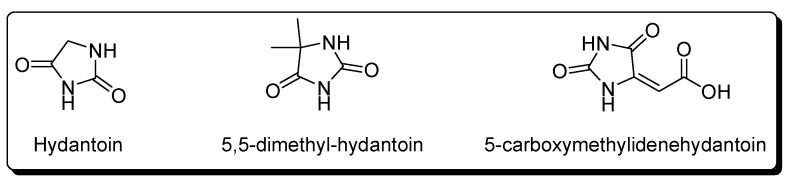
Hydantoins identified in the acid hydrolysate of the HCN oligomers.

Ferris *et al*. obtained hydantoin, 5,5-dimethyl-hydantoin and 5-carboxymethylidenehydantoin (Figure 2) using aqueous solutions of NaCN (1 M) at pH 9.2 (adjusted with HCl) with reaction times of several months at room temperature and after acid hydrolysis [81,85]. These compounds were identified using paper chromatography, thin layer chromatography and colorimetric methods.

These hydantoins are the cyclic products of the amino acids glycine, α-aminoisobutyric acid and diaminosuccinic acid (Table 1). One possible pathway for the formation of these hydantoins during acid hydrolysis is the cyclisation of the respective carbamyl amino acids. The isolation of urea from the oligomerisation mixture and citrulline (Table 1) from the acid hydrolysate of the HCN oligomers is consistent with the presence of carbamyl groups in the HCN oligomers [80,86].

## 3. Nucleic-Acid Bases

**Figure 3:**
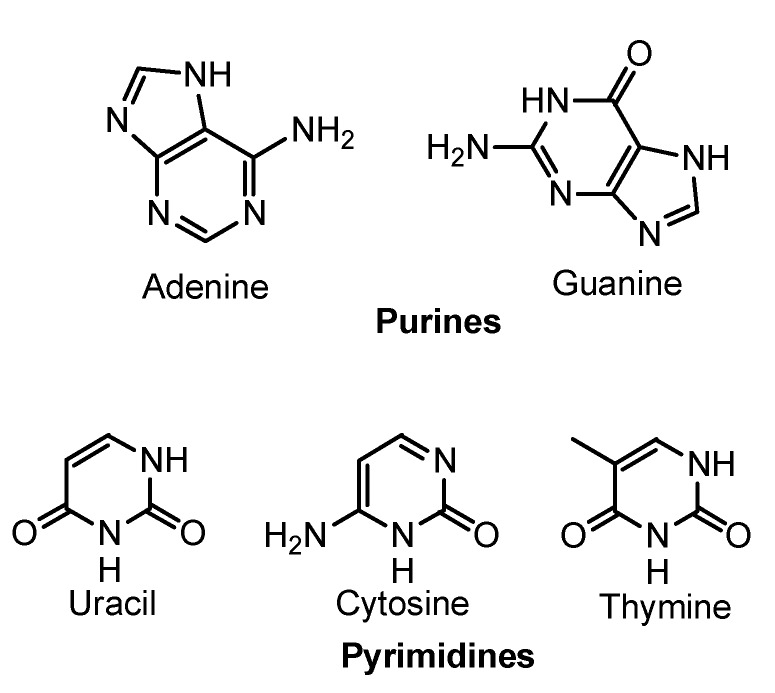
Nucleic-acid bases.

It is generally accepted that one of the principal characteristic of life is the ability to transfer information from one generation to the next. All modern living organisms have a genetic code for storing and transmitting information based on a chemical system of nucleic acids: DNA and RNA. The nucleic acids contain the keys to construct the enzymes via the process of protein synthesis. Since Gilbert’s hypothesis of an “RNA world” evolving at an early stage of life on primitive Earth, a variety of prebiotic syntheses have been proposed for the formation of the elementary building blocks of RNA and DNA: purine and pyrimine nucleic acid bases, ribose or 2'-deoxyribose, and phosphate [87,88]. Figure 3 shows the purines and pyrimidines present in the RNA and DNA (uracil in RNA and thymine in DNA). Today, it is well known that both RNA and DNA are able to carry out the catalytic activities required to start life on Earth [89,90].

### 3.1. Purines

The first prebiotic synthesis of purines was carried out by Oró [6] using concentrated aqueous solutions of ammonium cyanide, which were heating at 70 °C for several days. Adenine was identified in the raw reaction mixtures by the R_f_ values (paper chromatography) and the UV spectra against authentic standards. Pullman showed that the purine base adenine occupies a unique position in the purine family: in comparison to the other purines, it has the greatest resonance energy per π-electron and thus is likely to have been incorporated preferentially into biomolecules [91].

Two years after the first detection of adenine in Oró’s synthesis, Lowe *et al*. [77] also identified adenine and hypoxanthine in the total hydrolysed mixture from the heating of ammonium cyanide solution at 90 °C for 18 h. During the next decade, Ferris and co-workers obtained adenine using diluted solution of HCN (0.1 M) adjusted to pH 9.2 with NH_4_OH at room temperature for longer reaction times (4–12 months). Schwartz *et al*. explored in the 1980s the previous proposal made by Sanchez *et al*. [64,92]. Their results suggested that freezing dilute solutions of HCN in water could have provided conditions for the synthesis of bio-organic compounds in the primitive Earth. A freezing solution of HCN (0.01 M) was kept at −2 °C for 98 d (initial pH 9.2 adjusted with NH_4_OH). The acid hydrolysate yielded 0.004% adenine [93]. The addition of glyconitrile (the product of the addition of HCN and formaldehyde) to the reaction mixture under the same reaction conditions increased the yield of adenine to 0.02%. Indeed, Schwartz *et al*. demonstrated that formaldehyde, if introduced into solutions of HCN, could accelerate the oligomerisation of HCN under certain conditions [94]. The same research group demonstrated that unhydrolysed oligomerisation mixtures prepared from HCN in the absence of formaldehyde contain adenine-8-carboxamide, a possible precursor of adenine [95]. However, 8-hydroxymethyladenine is mostly formed in oligomerisation solutions of HCN when formaldehyde is added, rather than adenine-8-carboxamide or adenine itself [96]. Also in the context of a primitive cold Earth or icy satellites, Miller and co-workers demonstrated the formation of several purines (xanthine, hypoxanthine, adenine, guanine and 2,6-diaminopurine) in oligomerisation processes of HCN at temperatures between −20 and −78 °C [66,67].

Adenine was also tentatively identified in radiolysis experiments with a pure aqueous solution of HCN (pH 6) [97].

All purines detected in HCN polymers are shown in Table 2, together with the cyanide polymerisation reaction conditions and the analytical tools used for their identification. Interestingly, the hydrolysis conditions significantly influence the yields for each purine found. In this context, Ferris *et al*. [82], Miyakawa *et al*. [66] and Borquez *et al*. [98] reported interesting works about the influence of the hydrolysis conditions on the yield of purines and other organics released from HCN polymers.

Both AMN and DAMN were independently detected and isolated by Matthews and Moser [25] and by Sánchez *et al*. [64].

**Table 2:** Purines identified in Hydrogen Cyanide (HCN) polymers. The reaction conditions are described together with the material analysed: soluble fraction (solution or soluble oligomers), insoluble fraction (precipitated dark solid) or a combination of both. c (M) = initial molar concentration of the reactant in aqueous solution; HCN_(L)_ = HCN in liquid phase; HCN_(G)_ = HCN in gas phase; d = days; m = moths; y = years; (s) = saturated; Non hydrolysis = no additional hydrolysis (acid, basic or neutral) was made over the final product analysed; Acid = HCl 6 N/110 °C/24 h; Acid (2) = HCl 5 M/110 °C/18 h; Acid (3) = 98% HCOOH/170 °C/2 h; Neutral = phosphate 0.1 M (pH 8)/140 °C/3 days. Neutral (2) = HCl (pH 8.5)/110 °C/24 h; GC-MS = Gas Chromatography-Mass Spectrometry. PC = paper chromatography. TLC = thin layer chromatography. PE = paper electrophoresis; HPLC-UV = High performance liquid chromatography-UV detector; D = detected but not quantified; t = tentatively identified.

Compound	Starting material, c (M)	T (ºC)/t/Catalyser	Final product analysed	Hydrolysis	Yield (%)	Method of identification	Reference
Xhanthine	HCN, 0.1	−78/27 y/NH_3_ (pH 9.2)	Solution + black solid	Acid	0.022	HPLC-UV, GC-MS	[66]
				Neutral	0.022		
Hypoxanthine	HCN, 1.5	90/18 h/NH_3_	Solution + black solid	Acid	~1 μmol/L	PC, UV-spectrum, PE	[77]
	NaCN, 1	38/3–30 d/NH_4_Cl	Black solid	Acid	D	GC-MS	[46]
	HCN, 0.1	−78/27 y/NH_3_ (pH 9.2)	Solution + black solid	Acid	0.0041	HPLC-UV, GC-MS	[66]
				Neutral	0.0058		
Adenine	HCN, (s)	90/24 h/NH_4_OH (1.5 M)	Solution	Acid	D	PC, UV-spectrum	[6]
	HCN, 1.5	90/18 h/NH_3_	Solution + black solid	Acid	~1 μmol/L	PC, UV-spectrum, PE	[77]
	HCN, 9.9	90/8 d/NH_4_OH	Solution	Non hydrolysis	60 mg/L	2D-PC, UV-spectrum	[78]
	HCN, 10	80/24 h/NH_4_OH	Solution	Acid	0.027	HPLC-UV	[99]
	HCN, 1.5	70/2 d/NH4OH (3 M)	Solution	Non hydrolysis	D	2D-PC, UV-spectrum	[78]
	HCN, 11.1	70/5 d/NH_4_OH (12.8 M)	Solution	Non hydrolysis	110 mg/L	2D-PC, UV-spectrum	[78]
				Acid	700 mg/L		
	NaCN, 1	38/3–30 d/NH_4_Cl	Black solid	Acid	D	GC-MS	[46]
	HCN, 14.6	r.t./26 h/NH_4_OH (7 M)	Solution	Non hydrolysis	D	2D-PC, UV-spectrum	[78]
	HCN, 8.25	r.t./26 h/NH_4_OH (13 M)	Solution	Non hydrolysis	D	2D-PC, UV-spectrum	[78]
	HCN, 0.1	r.t./1 w/NH_4_OH (pH 9.2)	Solution + black solid	Acid	0.000013	HPLC-UV, GC-MS	[66]
	HCN, 0.1	r.t./4 w/NH_4_OH (pH 9.2)	Solution + black solid	Acid	0.00031	HPLC-UV, GC-MS	[66]
	HCN, 0.1	r.t./8 w/NH_4_OH (pH 9.2)	Solution + black solid	Acid	0.00062	HPLC-UV, GC-MS	[66]
	HCN, 0.1	r.t./4–12 m/NH_4_OH (pH 9.2)	Soluble oligomers	Acid	0.003–0.004	GC-MS	[82]
	NaCN, 1	r.t./1 y/pH 9.2 (HCl)	Soluble oligomers	Acid	D	TLC, UV-spectrum	[82]
	NaCN, 2 (+HCOH)	r.t./9 m/pH 9.2 (HCl)		Non hydrolysis	3 μmol/L	HPLC, UV spectrum, MS	[96]
				Neutral (2)	0.06		
	HCN, 0.2	r.t.−40 °C/γ-radiation/pH 6	Solution	Acid (3)	t	HPLC, GC-MS	[97]
	HCN, 0.01 (+glyconitrile)	−2/60 d/NH_4_OH (pH 9.2)	Solution	Acid (2)	0.02	HPLC-UV	[93]
	HCN, 0.01	−2/98 d/NH_4_OH (pH 9.2)	Solution	Acid (2)	0.004	HPLC-UV	[93]
	HCN, 0.1	−20/2 m/NH_3_ (pH 9.2)	Solution	Acid	0.005	HPLC-UV, ESI-MS	[67]
	HCN, 0.001	−20/3 m/NH_4_OH (pH 9.2)	Solution + black solid	Acid	0.0042	HPLC-UV, GC-MS	[66]
	HCN, 0.01	−20/3 m/NH_4_OH (pH 9.2)	Solution + black solid	Acid	0.01	HPLC-UV, GC-MS	[66]
	HCN	−20/3 m/NH_4_OH (pH 9.2)	Solution + black solid	Acid	0.0094	HPLC-UV, GC-MS	[66]
	HCN, 0.1	−20/25 y/NH_3_ (pH 9.2)	Solution	Acid	0.038	HPLC-UV	[99]
	HCN, 0.1	−20/25 y/NH_3_ (pH 9.2)	Solution	Acid	0.035	HPLC-UV, ESI-MS	[67]
	NaCN, 0.1	−30/2 m/NH_4_Cl	Solution	Acid	0.0004	HPLC-UV	[99]
	HCN, 0.1	−78/25 y/NH_3_ (pH 9.2)	Solution	Acid	0.04	HPLC-UV, ESI-MS	[67]
	HCN, 0.1	−78/27 y/NH_3_ (pH 9.2)	Solution + black solid	Acid	0.029	HPLC-UV, GC-MS	[66]
				Neutral	0.012		
				Non hydrolysis	0.00016		
Guanine	HCN, 10	80/24 h/NH_4_OH	Solution	Acid	0.0007	HPLC-UV	[99]
	NaCN, 1	38/3–30 d/NH_4_Cl	Black solid	Acid	D	GC-MS	[46]
	HCN, 0.1	−20/25 y/NH_3_ (pH 9.2)	Solution	Acid	0.0035	HPLC-UV	[99]
	HCN, 0.1	−20/25 y/NH_3_ (pH 9.2)	Solution	Acid	0.0004	HPLC-UV, ESI-MS	[67]
	NaCN, 0.1	−30/2 m/NH_4_Cl	Solution	Acid	0.000014	HPLC-UV	[99]
	HCN, 0.1	−78/27 y/NH3 (pH 9.2)	Solution + black solid	Acid	0.0067	HPLC-UV, GC-MS	[66]
				Neutral	0.0033		
				Non hydrolysis	0.00011		
2,6-diaminopurine	HCN, 0.1	−78/27 y/NH3 (pH 9.2)	Solution + black solid	Neutral	0.0091	HPLC-UV, GC-MS	[66]
8-hydroxymethyladenine	NaCN, 2 (+HCOH)	r.t./9 m/pH 9.2 (HCl)		Non hydrolysis	47 μmol/L	HPLC, UV spectrum, MS	[96]
				Neutral (2)>	0.06		

**Figure 4:**
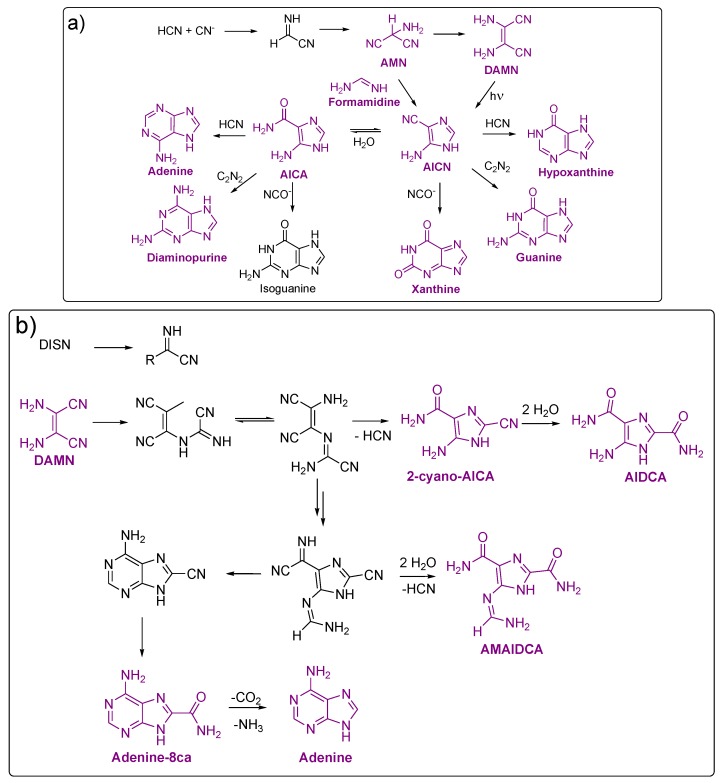
Proposed mechanisms for the formation of purines from HCN. (**a**) Ferris and Orgel; (**b**) Voet and Schwartz. AICA, 4[5]-aminoimidazole-5[4]-carboxamide; AICN, 4[5]-aminoimidazole-5[4]-carbonitrile; AIDCA, 4[5]-aminoimidazole-2,5[4]-dicarboxamide;AMAIDCA, 4[5]-*N*-(aminomethylidene)-aminoimidazole-2,5[4]-dicarboxamide; Adenine-8-ca, adenine-8-carboxamide. The purines and their precursors identified in the HCN polymers are marked in purple.

Oró proposed that the formation of adenine proceeded by the reaction of the HCN trimer (aminomalonitrile, AMN) with formamidine [78]. However, this mechanism was discarded because the hydrolysis of formamidine is very rapid and because the relative rate of reaction of cyanide with AMN is much greater than the rate of the reaction of formamide with AMN [64]. These results suggested that the conditions required for the formation of AMN will result in its rapid conversion to the tetramer (diaminomalonitrile, DAMN) by reaction with cyanide. Thus, DAMN or compounds derived from it must be precursors of adenine. This mechanistic proposal by Ferris and Orgel is shown in Figure 4a: AMN reacts to formamide to produce 4[5]-aminoimidazole-5[4]-carbonitrile (AICN), a compound that readily yields a variety of purines under plausible prebiotic conditions [100,101] This synthesis is not possible, as it is indicated above, in dilute solutions because of the competing hydrolysis of formamidine to formamide. On the other hand, independently of the formamidine concentration, Ferris and Orgel demonstrated the photochemical rearrangement of DAMN into AICN [100]. AICN and a product of its hydrolysis, 4[5]-aminoimidazole-5[4]-carboxamide (AICA), reacts with HCN, cyanate (NCO^−^), formamidine, or cyanogen (CN)_2_ to give adenine, hypoxanthine, diaminopurine, xanthine, isoguanine and guanine, respectively. Both imidazoles, AICA and AICN have been identified in HCN polymers [78,82].

Schwartz and Voet proposed a pathway from HCN to adenine that does not implicate AICN or AICA as an intermediate (Figure 4b). This mechanism is based on the isolation of adenine-8-carboxamide, which readily hydrolyses to adenine [95]. In this approach, the main precursor is also DAMN, a compound that is stable and present in appreciable concentration in an oligomerising solution of HCN [58]. The 2-substituted imidazole derivatives 2-cyano-AICA, 4[5]-aminoimidazole-2,5[4]-dicarboxamide (AIDCA) and 4[5]-*N*-(aminomethylidene)-aminoimidazole-2,5[4]-dicarboxamide (AMAIDCA) were isolated from HCN polymers using a solution of HCN (1 M, pH 9.2 adjusted with NH_4_OH) stored at room temperature for eight months. The 2-substituted imidazoles were identified before hydrolysis using spectroscopic techniques and mass spectrometry [95].

### 3.2. Pyrimidines

In the late 1970s, Ferris and co-worker identified pyrimidines for the first time (4,5-dihydroxypyrimidine, 5-hydroxyuracil and orotic acid) in the acid hydrolysates of HCN oligomers synthesised at room temperature [82,102]. Under similar polymerisation conditions for HCN, Voet and Schwartz identified uracil using a laborious purification work-up and hydrolysing HCN oligomers [103]. Under the simulation conditions of a cold Earth, Miyakawa *et al*. achieved the greatest diversity in pyrimidines (4,5-dihydroxypyridine, uracil, 5-hydroxyuracil, 5-aminouracil, orotic acid and 5-aminoorotic acid) [66]. Tentatively, Negrón-Mendoza *et al*. detected uracil, cytosine and thymine in experiments of radiolysis of HCN aqueous solutions [97]. In Table 3, all pyrimidines found in HCN polymers are summarised. As in the case of purines, the yield in pyrimidines released from HCN polymers depends strongly on the hydrolysis conditions assayed (see Table 3). Additionally, it is interesting to note that the development of analytical chromatography techniques has allowed the identification of a greater diversity of pyrimidines, as can be readily seen in the more recent works [46,66].

**Figure 5:**
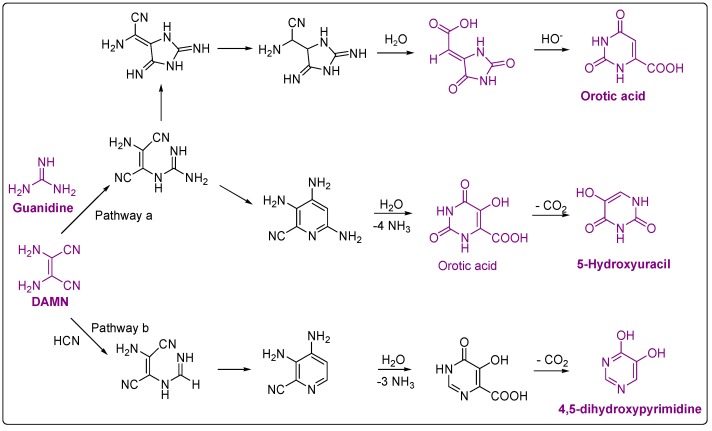
Proposal mechanisms for the formation of pyrimidines from HCN by Ferris *et al*. [102]. The compounds identified in the HCN polymers are marked in purple.

Ferris *et al*. proposed the following mechanism (Figure 5) to explain the formation of the pyrimidines identified by them, starting from the stable tetramer of HCN, DAMN [102]. 

**Table 3:** Pyrimides identified in HCN polymers. The reaction conditions are described together with the material analysed: soluble fraction (solution or soluble oligomers), insoluble fraction (precipitated dark solid) or a combination of both. c (M) = initial molar concentration of the reactant in aqueous solution; d = days; m = moths; y = years; Acid = HCl 6 N/110 °C/24 h; Non hydrolysis = no additional hydrolysis was made over the final product analysed; Acid (2) = HCl 5 M/110 °C/18 h; Acid (3) = 98% HCOOH/170 °C/2 h; Neutral = phosphate 0.01 M (pH 8)/140 °C/3 days; Neutral (2) = NaOH (pH 5)/110 °C/24 h; GC-MS = Gas Chromatography-Mass Spectrometry; TLC = thin layer chromatography; HPLC-UV = High performance liquid chromatography-ultraviolet detector; D = detected but not quantified; t = entatively identified.

Compound	Starting material, c(M)	T (ºC)/t/Catalyst	Final product analysed	Hydrolysis	Yield (%)	Method of identification	Reference
4,5-Dihydroxypyrimidine	HCN, 0.1	r.t./4–12 m/NH_4_OH (pH 9.2)	Soluble oligomers	Acid	0.62	TLC, UV spectrum	[102]
	HCN, 0.1	r.t./4–12 m/NH_4_OH (pH 9.2)	Soluble oligomers	Acid	0.7–0.9	GC-MS	[82]
	NaCN, 1	r.t./1 y/pH 9.2 (HCl)	Soluble oligomers	Acid	D	TLC, UV spectrum	[82]
	HCN, 0.1	−78/27 y/NH_3_ (pH 9.2)	Solution + black solid	Acid	0.65	HPLC-UV, GC-MS	[66]
Uracil	NaCN, 1	38/3–30 d/NH_4_Cl	Black solid	Acid	D	GC-MS	[46]
	HCN, 1	r.t./6 m/NH_4_OH (pH 9.2)	Solution	Acid (2)	0.001	HPLC-UV	[103]
	HCN, 0.1	r.t./6 m/NH_4_OH (pH 9.2)	Solution	Acid (2)	0.005	HPLC-UV	[103]
	NaCN, 1	r.t./6 m/pH 9.2 (HCl)	Solution	Acid (2)	0.001	HPLC-UV	[103]
	HCN, 0.1	−78/27 y/NH_3_ (pH 9.2)	Solution + black solid	Acid	0.00026	HPLC-UV, GC-MS	[66]
				Neutral	0.0017		
	HCN, 0.2	r.t.−40 °C/γ-rad (60Co)/pH 6	Solution	Acid (3)	t	HPLC, GC-MS	[97]
5-Hydroxyuracil	NaCN, 1	38/3–30 d/NH_4_Cl	Black solid	Acid	D	GC-MS	[46]
	HCN, 0.1	r.t./4–12 m/NH_4_OH (pH 9.2)	Soluble oligomers	Acid	0.002–0.004	TLC, UV spectrum	[102]
	HCN, 0.1	r.t./4–12 m/NH_4_OH (pH 9.2)	Soluble oligomers	Acid	0.003	GC-MS	[82]
	HCN, 0.1	−78/27 y/NH_3_ (pH 9.2)	Solution + black solid	Acid	0.0015	HPLC-UV, GC-MS	[66]
Cytosine	HCN, 0.2	r.t.−40 °C/γ-rad (^60^Co)/pH 6	Solution	Acid (3)	t	HPLC, GC-MS	[97]
5-Aminouracil	NaCN, 1	38/3–30 d/NH_4_Cl	Black solid	Acid	D	GC-MS	[46]
	HCN, 0.1	−78/27 y/NH_3_ (pH 9.2)	Solution + black solid	Acid	0.0058	HPLC-UV, GC-MS	[66]
				Neutral	0.0038		
Orotic acid	NaCN, 1	38/3–30 d/NH_4_Cl	Black solid	Acid	D	GC-MS	[46]
	HCN, 0.1	r.t./4–12 m/NH_4_OH (pH 9.2)	Soluble oligomers	Neutral (2)	0.009	TLC, UV spectrum	[102]
	HCN, 0.1	r.t./4–12 m/NH_4_OH (pH 9.2)	Soluble oligomers	Acid	0.009	GC-MS	[82]
	HCN, 0.1	−78/27 y/NH_3_ (pH 9.2)	Solution + black solid	Acid	0.0025	HPLC-UV, GC-MS	[66]
				Neutral	0.1		
5-Aminoorotic acid	HCN, 0.1	−78/27 y/NH_3_ (pH 9.2)	Solution + black solid	Neutral	0.019	HPLC-UV, GC-MS	[66]
	HCN, 0.1	−78/27 y/NH_3_ (pH 9.2)	Solution + black solid	Non hydrolysis	0.00028	HPLC-UV, GC-MS	[66]
Thymine	HCN, 0.2	r.t.−40 °C/γ-rad (^60^Co)/pH 6	Solution	Acid (3)	t	HPLC, GC-MS	[97]
1,2,5,6-Tetrahydropyrimidine	NaCN, 1	38/3–30 d/NH_4_Cl	Black solid	Acid	D	GC-MS	[46]

## 4. Carboxylic Acids

As we can see, the above HCN polymers contain the chemical information for the synthesis of nucleic-acid bases and have been used as macromolecular precursors for both purine and pyrimidine derivatives upon acidic or basic hydrolysis and as amino acids. Moreover, the HCN polymers also present the ability to release carboxylic acids after acid hydrolysis. 

Several monocarboxylic acids have been detected in HCN polymers. Lowe *et al*. 1963 [77] identified formic acid in an ether extract of a dried portion of the total reaction mixture in an experiment at a high polymerisation temperature. In an experiment with moderate conditions, glycolic acid was identified using GC-MS in the black insoluble polymer, after acid hydrolysis [46]. The radiolysis of HCN using a 60Co source led to the production of butyric acid [104].

**Figure 6:**
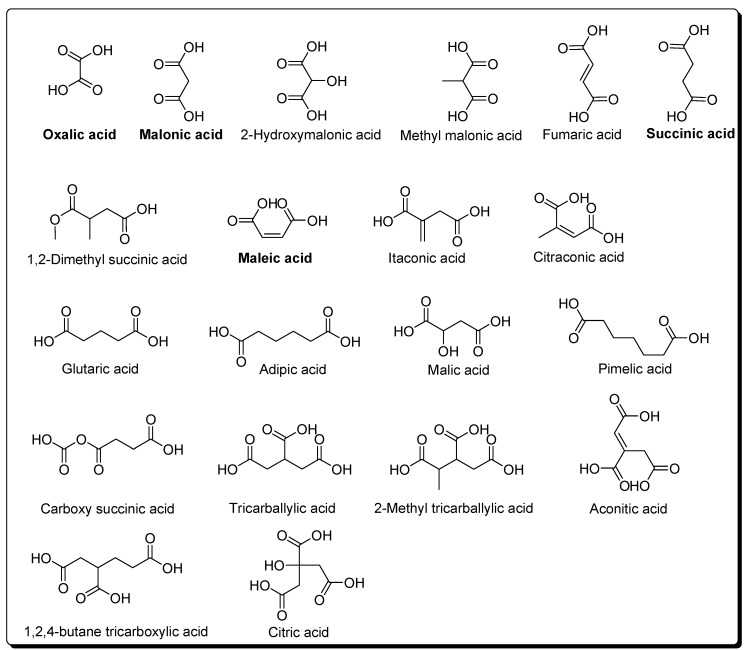
Di- and tricarboxylic acids produced by radiolysis of HCN. In bold are marked the acids also obtained from polymerisation of NH_4_CN.

In this same radiolysis experiment, a large set of dicarboxylic and tricarboxylic acids were identified as their methyl-ester derivatives extracted with water-benzene using GC-MS [104] (Figure 6). This experiment was also reproduced in the presence of clays, and again a set of carboxylic acids was obtained [105]. The production of carboxylic acids is a function of the radiation dose, and the presence of clays led to lower production. The proposed mechanism for the formation of carboxylic acids in radiolysis experiments of HCN is the following:

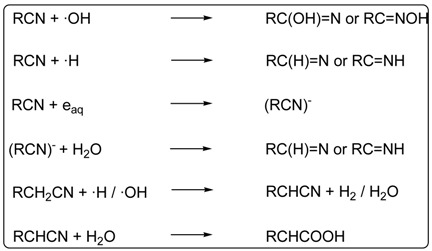



Oxalic acid is largely known as a polymerisation product of HCN in aqueous solutions [81]. However, it has recently been demonstrated that the formation of other dicarboxylic acids is possible in NH4CN polymerisation processes. The acid hydrolysates of black HCN polymers yielded oxalic acid, malonic acid, 2-hydroxymalonic acid, succinic acid and maleic acid [46].

**Figure 7:**
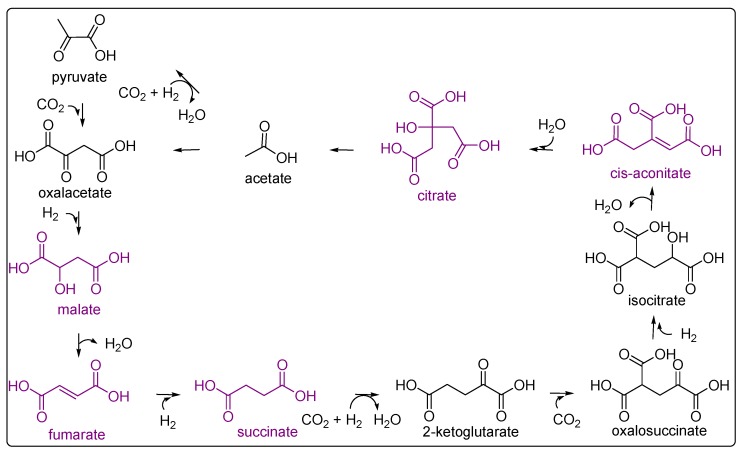
The carboxylic acids detected in the HCN polymers that are related to a plausible reductive Krebs cycle are marked in purple. The scheme is adapted from Smith and Morowitz [107].

The identification of di- and tricarboxylic acids from HCN polymerisation is very interesting from the perspective of the emergence of a primordial metabolic cycle. Eschenmoser suggested that a relationship exists between HCN and the constituents of the reductive citric acid cycle [106]. The acids implicated in a plausible inverse Kreb’s cycle and the di- and tricarboxylic acids identified in HCN polymers are shown in Figure 7.

## 5. Carbonyl Compounds

**Figure 8:**
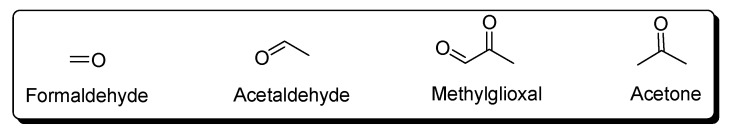
Aldehydes and ketones obtained in radiolysis experiments of aqueous HCN solutions.

In the radiolysis experiments of aqueous solutions of HCN in the absence or presence of NH3 (pH 6 and 9, respectively), several aldehydes and ketones were identifiable in the crude reaction mixtures (Figure 8) using GC-FID [104].

## 6. Pteridines

Recently, Ruiz-Bermejo *et al*. [21] identified a pteridine, 2,4,7-trihydroxy-pteridine, during preliminary analysis of the acid hydrolysate of black HCN polymers using GC-MS. These results are interesting because this compound can be related to several cofactors, such as riboflavins and pterins. Moreover, the cofactors or coenzyme structures are considered “molecular fossils” of an early phase of life, and a hydrocyanic origin of cofactor building blocks has been suggested [1,108,109,110].

## 7. Others

**Figure 9:**
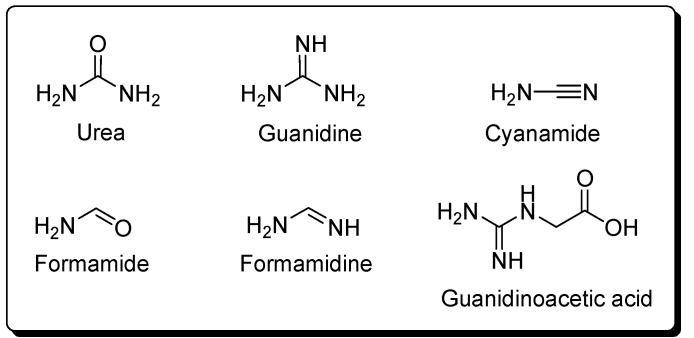
Urea and related compounds identified in the reaction mixtures of the HCN polymers.

Urea is a principal product of the polymerisation reactions of HCN in aqueous solutions. It can be identified before hydrolysis of the crude reaction mixtures [72,77,81,82]. Lowe *et al*. [77] estimated a yield of 0.16 mol/L in heating experiments for the polymerisation of HCN. The hydrolysis product of urea, formamide, and formamidine can also be identified [78] together with other urea related compounds, such as guanidine [72,82], guanidinoacetic acid [72,82] and cyanamide [111] in HCN polymers. These compounds were identified by their R_f_ values against authentic standards and by colorimetric methods (Figure 9).

## 8. Summary

**Figure 10:**
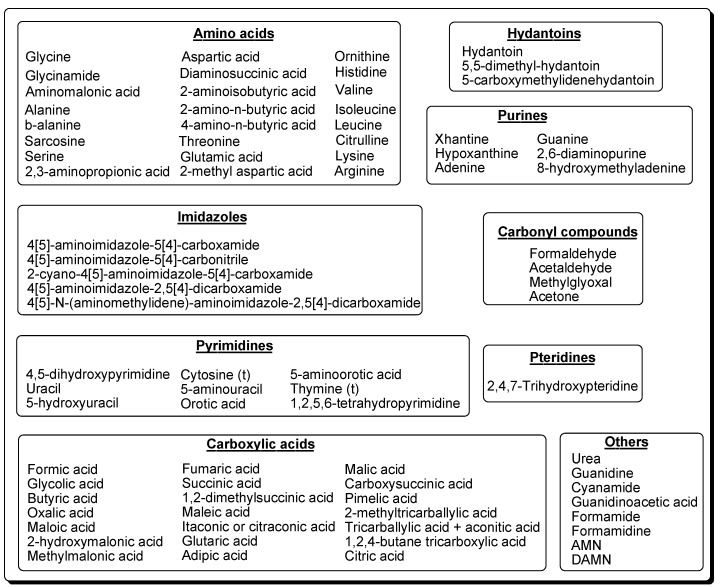
Summary of the simple organics and biomonomers identified in HCN polymers.

HCN polymers can be obtained under a wide variety of conditions. Depending on the polymerisation conditions, the treatment of the raw reactions and/or the analytical tools, a great diversity of simple organics and biomonomers can be detected. In HCN polymers, amino acids, purines, pyrimidines, carboxylic acids, aldehydes and ketones, pteridines, urea and urea-related compounds have been identified (Figure 10). Many of these organics compounds are building blocks of current proteins and nucleic acids or are active participants in metabolism. Therefore, the HCN polymers play a central core in a plausible protobiological system.
